# Interferon Regulatory Factor-8 Is Important for Histone Deacetylase Inhibitor-Mediated Antitumor Activity

**DOI:** 10.1371/journal.pone.0045422

**Published:** 2012-09-19

**Authors:** Debarati Banik, A. Nazmul H. Khan, Even Walseng, Brahm H. Segal, Scott I. Abrams

**Affiliations:** 1 Department of Immunology, Roswell Park Cancer Institute, Buffalo, New York, United States of America; 2 Department of Medicine, Roswell Park Cancer Institute, Buffalo, New York, United States of America; 3 Department of Immunology, Institute for Cancer Research, Oslo University Hospital, Oslo, Norway; National Cancer Institute (INCA), Brazil

## Abstract

The notion that epigenetic alterations in neoplasia are reversible has provided the rationale to identify epigenetic modifiers for their ability to induce or enhance tumor cell death. Histone deacetylase inhibitors (HDACi) represent one such class of anti-neoplastic agents. Despite great interest for clinical use, little is known regarding the molecular targets important for response to HDACi-based cancer therapy. We had previously shown that interferon regulatory factor (IRF)-8, originally discovered as a leukemia suppressor gene by regulating apoptosis, also regulates Fas-mediated killing in non-hematologic tumor models. Furthermore, we and others have shown that epigenetic mechanisms are involved in repression of IRF-8 in tumors. Therefore, in our preclinical tumor model, we tested the hypothesis that IRF-8 expression is important for response to HDACi-based antitumor activity. In the majority of experiments, we selected the pan-HDACi, Trichostatin A (TSA), because it was previously shown to restore Fas sensitivity to tumor cells. Overall, we found that: 1) TSA alone and more so in combination with IFN-γ enhanced both IRF-8 expression and Fas-mediated death of tumor cells in vitro; 2) TSA treatment enhanced IRF-8 promoter activity via a STAT1-dependent pathway; and 3) IRF-8 was required for this death response, as tumor cells rendered IRF-8 incompetent were significantly less susceptible to Fas-mediated killing in vitro and to HDACi-mediated antitumor activity in vivo. Thus, IRF-8 status may underlie a novel molecular basis for response to HDACi-based antitumor treatment.

## Introduction

It is now widely accepted that both genetic and epigenetic alterations contribute to tumor initiation and progression [1]–[4]. Epigenetic gene repression, particularly of tumor suppressor genes, may occur via several reversible mechanisms, namely DNA methylation, histone deacetylation or a combination of both [1]–[4]. Hypomethylating agents, such as 5-aza-2′-deoxycytidine, or histone deacetylase inhibitors (HDACi), such as depsipeptide (DP), are being evaluated in cancer clinical trials [5]–[8]. Such epigenetic-based therapies have in common their ability to alter gene expression that facilitates tumor growth arrest or apoptosis [3], [7]–[9]. Despite great interest in their clinical use, little is known regarding molecular targets important for response to HDACi-based cancer therapy. Identification of HDACi targets, therefore, may lead to the discovery of new biomarkers of disease status, improve the way patients are selected for HDACi-based therapy and potentially guide the development of new drugs.

The loss of Fas function in neoplastic cells is thought to be an important mechanism both for resistance to certain chemotherapeutic agents and for tumor escape from immune attack [10]–[15]. Our earlier work led to the identification of interferon regulatory factor-8 (IRF-8) as a positive regulator of response to Fas-mediated killing of non-hematopoietic tumor cells [16], [17]. We further observed that low levels of both Fas and IRF-8 expression by tumor cells correlated with more rapid tumor growth [16], [17]. These data suggested that IRF-8 down-regulation (at least in certain cancers) contributes to tumor progression via increased resistance to apoptosis, such as Fas-mediated killing. Although IRF-8 was originally discovered as an IFN-γ inducible transcription factor essential for normal myelopoiesis [18], [19] and as a tumor suppressor of certain leukemias [18], [20]–[25], our findings revealed a new functional role for IRF-8 in non-hematopoietic malignancies. However, the mechanisms involved in IRF-8 down-regulation in tumor cells remained unclear. We reasoned that rescue of IRF-8 expression in tumor cells may improve responses to anti-neoplastic therapies, such as chemotherapy or biologic (Fas)-based immunotherapy.

Several studies now demonstrate that IRF-8 expression in various human cancers and tumor cell lines can be down-regulated by epigenetic mechanisms [17], [21], [26]–[29]. It has also been shown that Trichostatin A (TSA), a potent pan-HDACi, can reinstate Fas sensitivity in tumor cells [30], [31]. However, the molecular mechanisms for HDACi-induced apoptosis of tumor cells are not well-defined. We hypothesized that IRF-8 expression in tumor cells is an important molecular component for their susceptibility to HDACi-induced apoptosis. To test our central hypothesis, we focused on two questions: 1) Is IRF-8 expression in tumor cells required for their susceptibility to Fas-mediated killing induced by HDACi? and 2) Is IRF-8 expression required for HDACi to promote antitumor effects in tumor-bearing mice? Overall, our data show that HDACi enhances IRF-8 expression in tumor cells involving STAT1, and promotes Fas-mediated killing and antitumor activity via an IRF8-dependent pathway. Therefore, IRF-8 expression in tumors may represent a unique molecular marker for predicting response to HDACi-based therapies.

## Results

### HDAC Inhibitors Enhance IRF-8 Expression in Tumor Cells

**Figure 1:**
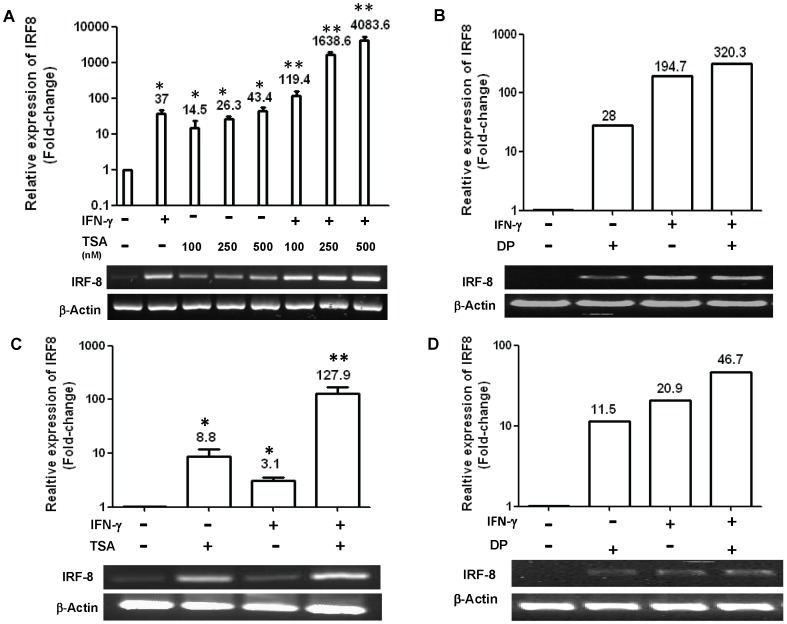
HDACi enhances IRF-8 expression in tumor cells. (*A*) CMS4 cells were treated with TSA, IFN-γ (100 U/ml) or a combination of both at the indicated concentrations and then analyzed by real-time PCR (top). Representative RT-PCR is shown in the bottom panel, which shares the same treatment labels. Data in top panel are presented as fold-change (shown above each bar) of the treated samples relative to the vehicle-treated controls. (*B*) Similar to *A*, except that CMS4 cells were treated with DP (25 ng/ml) instead of TSA. (*C*) CMS4-met.sel cells were treated with TSA (500 nM), IFN-γ or a combination of both and then analyzed real-time PCR (top) or RT-PCR (bottom), as in *A*. (*D*) Similar to *C*, except that CMS4 cells were treated with DP instead of TSA. All data are expressed as the mean ± SEM of triplicate determinations (shown above each bar). *P<0.05, based on comparing the single agent treatment to the vehicle-treated control. **P<0.05, based on comparing the combination regimen to the single treatment counterparts.

We first evaluated whether HDACi affects tumor cell expression of IRF-8. The effects of two HDACi on IRF-8 expression in tumor cells were studied in vitro: TSA, a well-studied experimental pan-HDACi [9], [30] and DP, which is currently being tested in cancer clinical trials [7], [8]. First, we treated CMS4 cells with IFN-γ, TSA or a combination of TSA and IFN-γ (Fig. 1A). As expected, IFN-γ significantly enhanced IRF-8 mRNA levels. TSA treatment (100–500 nM) also significantly enhanced IRF-8 expression in a dose-dependent fashion. Moreover, the level of IRF-8 expression after the combination treatment (TSA with IFN-γ) ranged from 119–4084-fold higher compared to untreated cells and was significantly higher than either treatment alone (Fig. 1A). We then extended this analysis to DP, a second HDACi (Fig. 1B). Similar to that seen with the TSA studies (Fig. 1A), DP treatment also enhanced IRF-8 expression levels. The combination treatment further enhanced IRF-8 levels, suggesting that DP, as with TSA, rendered CMS4 cells more receptive to IRF-8 induction by IFN-γ.

We next examined the effects of TSA or DP on IRF-8 expression using a highly aggressive metastatic variant of CMS4 cells, termed CMS4.met.sel [32]. This subline was established as a tumor escape variant following CD8^+^ CTL adoptive immunotherapy. Immune resistance correlated with a significant reduction in both Fas and IRF-8 expression in response to IFN-γ [32]. Here, we employed this subline to further explore the relationship between tumor phenotype and IRF-8 responsiveness, but this time in response to HDACi. As with CMS4 cells (Fig. 1A), treatment of CMS4.met.sel cells with either IFN-γ, TSA (500 nM) or DP led to a significant increase in IRF-8 expression (Fig. 1C and D). IRF-8 induction was further boosted when TSA or DP was combined with IFN-γ. It is important to note that while both cell lines were responsive to IRF-8 induction, the magnitude of these responses were substantially lower in CMS4.met.sel cells compared to CMS4 cells. Thus, in this cell line model of varying tumor aggressiveness, IRF-8 response to a single or combination HDACi-based treatment regimen correlated with tumor phenotype.

**Figure 2:**
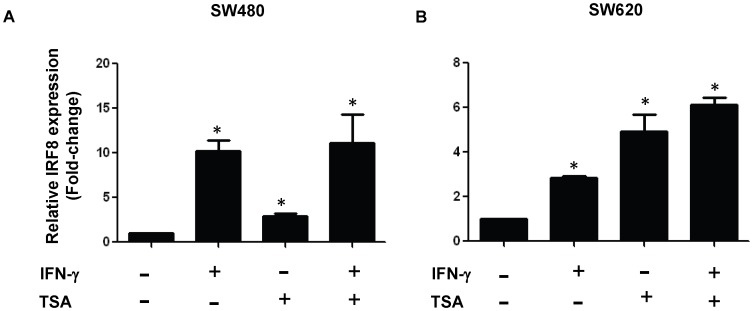
TSA enhances IRF-8 expression in a human tumor cell line model of varying malignant potential. SW480 (*A*) or SW620 (*B*) cells were treated with TSA (500 nM), IFN-γ (100 U/ml) or a combination of both and then analyzed by real-time PCR, as in Fig. 1. Data in *B* are presented as fold-change of the treated samples relative to the vehicle-treated controls. Data expressed as the mean ± SEM of triplicate determinations. *P<0.05, based on comparing the single agent treatment to the vehicle-treated control. **P<0.05, based on comparing the combination regimen to the single treatment counterparts.

Next, we extended our analysis to a second tumor cell line pair (Fig. 2). To do so, we made use of a human colon carcinoma cell line pair, SW480 and SW620, which like the CMS4 model, varies in malignant phenotype. SW480 and SW620 represent primary and metastatic cell lines, respectively, previously established from the same patient without any known prior systemic therapies [33]. And, as with the CMS4 model, we found that single agent or combination treatment enhanced IRF-8 expression in both cell lines (Fig. 2). Moreover, the magnitude of IRF-8 enhancement was greater in the primary tumor compared to the metastatic tumor, which also mirrored what we observed in the CMS4 system (Fig. 1). Under all treatment conditions and, in both cell line models minimal cellular toxicity (<10%) was observed, as detected by trypan blue dye exclusion. Taken collectively, these results show that HDACi can enhance basal or IFN-γ-inducible IRF-8 levels in tumor cell line models of varying malignant phenotypes and raise the possibility that HDACi may exert antitumor effects, at least in part, through IRF8-dependent mechanisms.

### TSA Treatment Facilitates Fas-mediated Killing via an IRF8-dependent Mechanism

Previously, we showed that IRF-8 expression in the CMS4 tumor model is required for Fas-mediated death, particularly in response to IFN-γ sensitization [16], [17]. Moreover, HDACi has been shown to restore Fas-mediated apoptosis in other tumor cell models via histone acetylation [30]. Thus, we sought to determine whether IRF-8 expression is required for Fas-mediated death in response to HDACi treatment. To that end, we made use of two distinct IRF-8 loss-of-function approaches, one based on RNA interference and the other based on ectopic dominant-negative expression. Although DP and TSA each induced IRF-8 expression in CMS4 tumor cells (Fig. 1), TSA was selected for subsequent experiments based on earlier work that showed that TSA treatment could restore Fas sensitivity in tumor cells [30].

**Figure 3:**
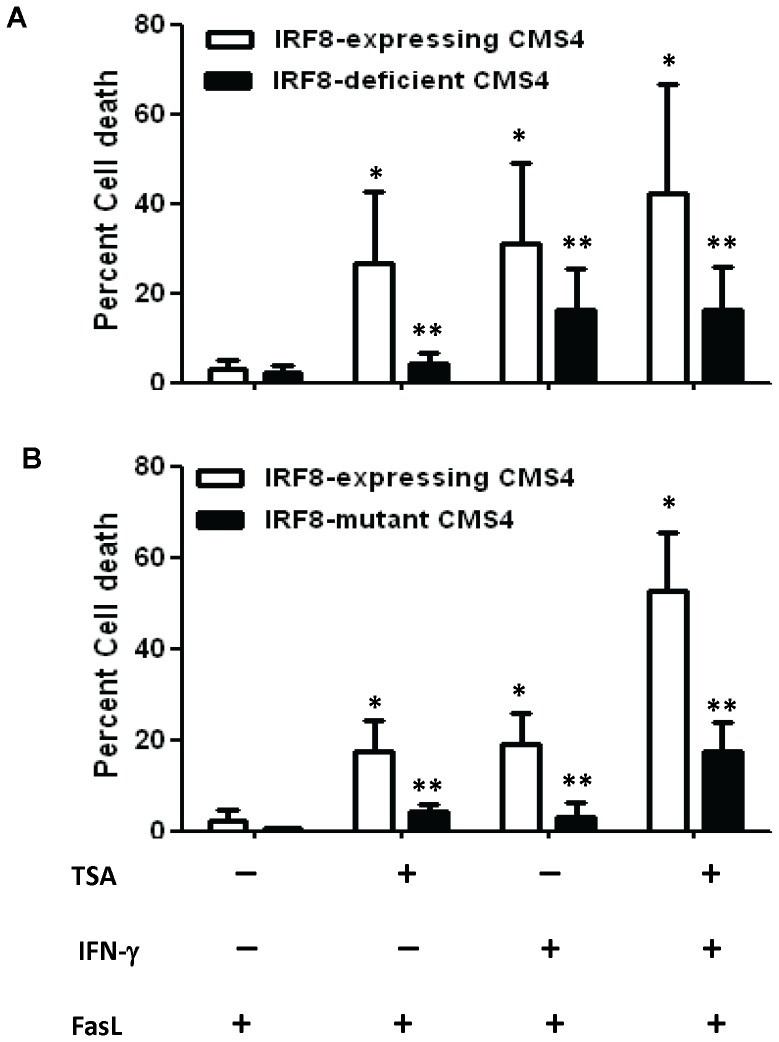
TSA treatment enhances Fas-mediated tumor cell death through an IRF-8-dependent mechanism. (*A*) Control or IRF-8-deficient CMS4-shRNA cells were exposed to recombinant mouse FasL (100 ng/ml) after treatment with TSA (100 nM), IFN-γ (200 U/ml), a combination of both or a vehicle control, and cell death measured by a flow-based assay. (*B*) IRF-8-mutant CMS4-K79E and CMS4-vector control cells were exposed to FasL after treatment with TSA (20 nM) and/or IFN-γ as in *A*. Data in *A* and B are expressed as mean ± SEM of six or three independent experiments, respectively. *P<0.05, based on comparing the indicated treatment group to the FasL only control. **P<0.05, based on comparing the IRF8-deficient to its matched IRF-8-expressing vector controls.

We compared CMS4 cells stably silenced for IRF-8 expression (i.e., CMS4-shRNA) to CMS4 cells stably transfected with a scrambled construct as a vector control, as previously reported [16]. We showed that treatment of the vector control cells with TSA, IFN-γ or a combination of both led to a significant increase in Fas-mediated death compared to vehicle-treatment conditions (Fig. 3A). Importantly, CMS4-shRNA cells were significantly less sensitive to Fas-mediated death compared to the vector control cells under these same treatment conditions (Fig. 3A). The TSA concentration (100 nM) chosen for these experiments was still capable of boosting IRF-8 expression in control, but not in CMS4-shRNA cells (data not shown). To strengthen these results, we employed a second approach to disrupt IRF-8 function. CMS4 cells were stably transfected with an expression plasmid encoding a mutant IRF-8 protein, termed K79E [34]. Previously, we showed that CMS4-K79E cells displayed a significant loss of Fas sensitivity [17]. Consistent with what we observed by RNA interference (Fig. 3A), CMS4-K79E cells were significantly less sensitive to Fas-mediated killing compared to the vector control cells in response to TSA and/or IFN-γ sensitization (Fig. 3B). Thus, under these conditions, these data indicate that TSA-induced Fas-mediated cell death is IRF-8-dependent.

### TSA Enhances IRF-8 Expression in a STAT1-dependent Manner

**Figure 4:**
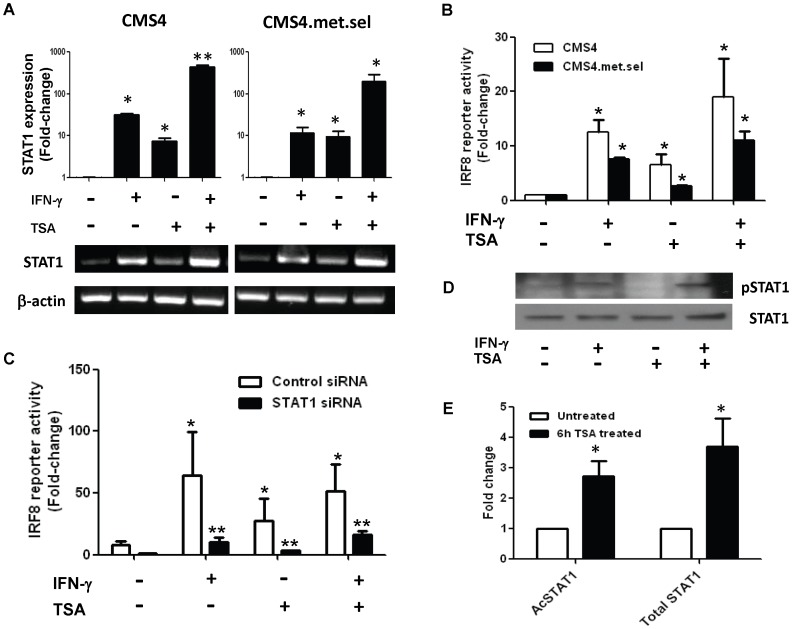
TSA-mediated IRF-8 transcription is STAT1-dependent. (*A*) STAT1 mRNA levels in CMS4 or CMS4.met.sel cells after the indicated treatments, as in Fig. 1. *(A)* Top, real-time PCR. Data presented as fold-change, as in Fig. 1. (*A*) Bottom, RT-PCR. *P<0.05, based on comparing the single agent treatment to the vehicle-treated control. **P<0.05, based on comparing the combination regimen to the single treatment counterparts. (*B*) CMS4 or CMS4.met.sel cells were transfected with an IRF-8 promoter reporter construct, followed by treatment with the indicated agents for 6 hr. Results are reported as the mean ± SEM of the fold-change relative to the vehicle-treated cells from three separate experiments. *P<0.05, based on comparing treatment to matched vehicle control. No activity was observed using the pGL3 vector lacking a promoter. (*C*) Similar to *B*, except that CMS4 cells were silenced for STAT1 expression. *P<0.05, based on comparing the indicated treatment group to the matched vehicle-treated control. **P<0.05, based on comparing the STAT1-deficient groups to their matched STAT1-expressing vector controls. (*D*) Phosphorylated STAT1 (pSTAT1) and total STAT1 protein levels in CMS4.met.sel cells after treatment with the indicated treatments (TSA, 500 nM; IFN-γ, 200 U/ml) for 15 min, as measured by Western blot. This experiment is representative of one of three with similar results. (*E*) Similar to *D*, except that acetylated STAT1 and total STAT1 levels were measured by IP-Western blot (i.e., IP with anti-STAT1 antibody, followed by Western blot with anti-acetyl-lysine antibody) after treatment with or without TSA (500 nM for 6 hr). Band intensities were quantified, and the data presented as fold-change of TSA-treated vs. untreated samples (mean ± SEM of triplicate experiments). *P<0.05, based on the TSA-treated group relative to the matched vehicle-treated control.

Janus-activated kinase-signal transducer and activator of transcription (JAK-STAT) pathways, specifically STAT1, play critical roles in the regulation of IFN-γ-inducible genes, including IRF-8 [19], [35]. To determine the role of STAT1 in TSA-mediated IRF-8 enhancement, we measured STAT1 transcript levels in both parental CMS4 and CMS4.met.sel cells after treatment with TSA, IFN-γ or both. First, we showed that IFN-γ treatment enhanced STAT1 mRNA levels in both cell lines (Fig. 4A). Secondly, TSA treatment alone and even more so in combination with IFN-γ increased total STAT1 mRNA levels in both cell lines. These data suggested that STAT1 expression was not compromised in either cell line.

To verify that events upstream of IRF-8 are intact in both cell lines, we made use of IRF-8 promoter reporter assays. CMS4 or CMS4.met.sel cells were transiently transfected with a luciferase reporter construct under the control of a bioactive IRF-8 promoter fragment, followed by the different treatments. Single agent IFN-γ or TSA treatment significantly increased IRF-8 promoter activity in both cell lines (Fig. 4B), reflecting their IRF-8 mRNA patterns (Fig. 1). To demonstrate the involvement of STAT1 in TSA-induced IRF-8 promoter activity, we measured luciferase activity in CMS4 cells transiently silenced for STAT1 expression. We found that TSA-induced IRF-8 promoter activity was significantly reduced in CMS4 cells silenced for STAT1 compared to the vector control (Fig. 4C). Similar patterns were observed in response to IFN-γ treatment or the combination treatment (Fig. 4C). In addition, we observed that STAT1 siRNA, but not the control sequence, blocked IFN-γ-inducible STAT1 as well as IRF-8 expression levels in both cell lines (data not shown). These data indicate that TSA or IFN-γ treatment can boost IRF-8 promoter activity via a STAT1-dependent mechanism.

To determine whether TSA-induced IRF-8 promoter activity functioned through STAT1 phosphorylation, we examined changes in phosphorylated STAT1 protein levels by Western blot analysis (10–120 min post-treatment). Whereas, IFN-γ or TSA in combination with IFN-γ led to detectable STAT1 phosphorylation in CMS4.met.sel cells compared to untreated cells, TSA treatment alone was unable to do so (Fig. 4D; shown at 15 min post-treatment; shorter or longer incubation times did not change outcome). Total STAT1 protein levels, however, were comparable among the different treatment groups. Similar results were observed in parental CMS4 cells in response to the different treatments (data not shown), indicating that the lack of TSA-induced STAT1 phosphorylation did not reflect subline-specific differences. These results indicate that the ability of TSA to enhance IRF-8 promoter activity is STAT1-dependent (Fig. 3C); albeit, it does not coincide with STAT1 phosphorylation status (Fig. 4D). These data are consistent with the ability of TSA to affect STAT1 activity via unphosphorylated-based mechanisms, such as acetylation [36]–[40]. To explore that possibility, the experiment was repeated and the lysates examined for STAT1 acetylation via IP for total STAT1 protein, followed by Western blot for acetylated lysine residues on STAT1. Importantly, we showed that TSA treatment led to a significant increase in acetylated STAT1 levels compared to the vehicle-treated control preparation (Fig. 4E). Furthermore, TSA treatment led to a significant increase in total STAT1 protein compared to the vehicle-treated control, which is consistent with the effect of TSA on STAT1 mRNA levels (Fig. 4A&E).

### TSA-mediated Antitumor Effects Require IRF-8 Expression

**Figure 5:**
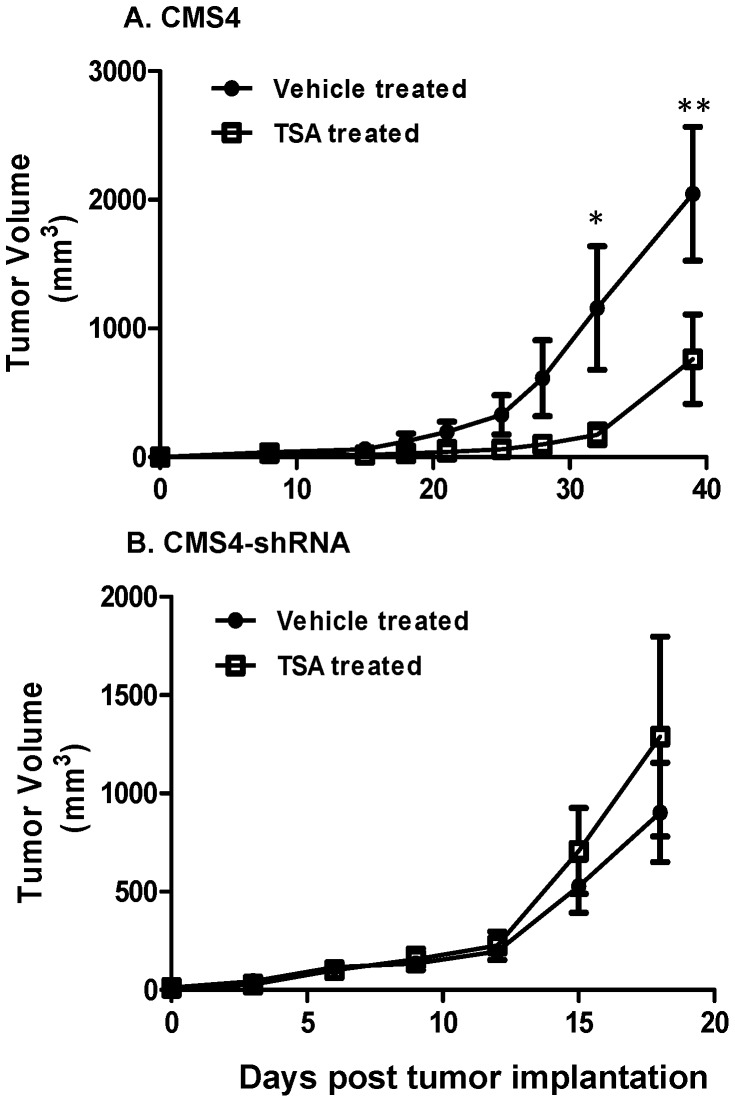
TSA-mediated antitumor activity requires tumor expression of IRF-8. CMS4 (*A*) or IRF-8-deficient CMS4-shRNA (*B*) tumor cells were injected SQ into BALB/c mice. When tumor size was palpable (∼30 mm^3^), mice were treated with six daily peritumoral injections of TSA or vehicle control. * *P*<0.01 or ** *P*<0.001, based on comparing the control to TSA-treated mice at the indicated time points in panel *A*. Data were not significant in panel *B*.

Our data indicate TSA treatment in vitro can facilitate Fas-mediated killing via an IRF8-dependent mechanism. Moreover, it has been shown that TSA, depending upon drug dose, can mediate antitumor activity in vivo [30], [41]. Thus, we hypothesized that tumor-cell expression of IRF-8 is also important for response to TSA-mediated antitumor activity in vivo. To test this hypothesis, we investigated the effects of TSA treatment in mice bearing either IRF8-competent (CMS4) or IRF8-deficient (CMS4-shRNA) tumor cells (Fig. 5). The schema involved several daily peritumoral injections of TSA to mice once tumors became palpable. We showed that TSA treatment of mice bearing IRF8-competent tumor cells led to dramatic tumor growth inhibition (Fig. 5A), suggesting that this TSA-based schema can indeed facilitate antitumor activity in vivo. In contrast, we showed that TSA treatment of mice bearing the IRF8-deficient tumor cells failed to promote significant antitumor effects (Fig. 5B), suggesting that ‘tumor response to therapy’ in vivo was IRF8-dependent.

## Discussion

Epigenetic modifiers, such as HDACi, have achieved encouraging results in both hematologic and non-hematologic cancer clinical trials [2], [7], [8]. Understanding key molecular features for response to such systemic therapies is critical to improving the way disease status is monitored and potentially how patients are selected for treatment. Since HDACi generally impact the expression of numerous genes [2], [7], [8], [42], it becomes difficult to determine which ones are relevant for ‘response to therapy’. Here, we took a more focused approach to elucidate molecular determinants required for HDACi-mediated antitumor effects.

Our model focused on Fas-induced death in response to HDACi treatment. Based on previous work that established that HDACi can enhance Fas sensitivity [30] and that IRF-8 expression was required for response to Fas killing [16], [17], we tested the hypothesis that tumor-cell expression of IRF-8 was required for Fas-induced death following HDACi treatment. We demonstrated that loss of IRF-8 expression led to a concomitant loss of Fas sensitivity to TSA-treated tumor cells in vitro. Moreover, we showed that TSA-mediated suppression of tumor growth in vivo was dependent on tumor expression of IRF-8. These new findings extend our previous work showing that tumor cell susceptibility to Fas-based effector mechanisms was IRF-8-dependent in vivo. Indeed, in mice lacking functional FasL expression, both control and IRF-8-deficient tumors grew at similar rates, whereas in wild-type mice, IRF-8-deficient tumors grew at a significantly higher rate than control tumors [16]. Together, these results indicate that HDACi promote Fas-mediated tumor cell death, in part, through IRF-8-dependent pathways.

It is likely that response to TSA in vivo involves a complex set of host-dependent and tumor-dependent interactions that require further elucidation. Nonetheless, it is important to emphasize that tumor-cell expression of IRF-8 was crucial for therapeutic response to HDACi. We also showed that TSA in combination with IFN-γ boosted IRF-8 expression. Similar results were observed with DP, suggesting that modulation of IRF-8 expression was not limited to TSA. Moreover, similar results with TSA were observed in a second tumor cell model, suggesting that the effects of HDACi on IRF-8 expression were not tumor model-specific. These results support the notion that HDACi, potentially in concert with certain innate or adaptive inflammatory signals, can enhance sensitivity to apoptosis in otherwise refractory or resistant tumor subpopulations [43]. The ability to do so was illustrated using a highly aggressive CMS4 subline, which became more responsive to IRF-8 induction following exposure to TSA or DP alone or in combination with IFN-γ. Although it remains to be fully investigated why the two cell lines varied in their response to IRF-8 induction, these data nonetheless provide evidence that IRF-8 is a key component for response to HDACi. Future studies will also determine whether the epigenetic profile of the IRF-8 promoter is different in CMS4 vs. CMS4-met.sel cells (or SW480 vs. SW620 cells), which may help to explain in part their differential responsiveness of IRF-8 induction to TSA treatment.

To further demonstrate the importance of IRF-8 in this model, we examined the effects of TSA on IRF-8 promoter activity using a reporter assay. It is important to note that this IRF-8 promoter construct contains the endogenous DNA sequence without any hypermethylation or HDAC sites. Thus, these experiments were designed not only to substantiate the effect of TSA on IRF-8 expression, but also to determine whether the effect of TSA on IRF-8 promoter activity was HDAC-dependent. We hypothesized that if the acetylation status of IRF-8 matters for response to TSA, then an IRF-8 promoter sequence lacking HDAC sites would be unresponsive to TSA treatment. We found that TSA alone and more so in combination with IFN-γ increased IRF-8 promoter activity in both parental and aggressive CMS4 cells. For both cell lines after TSA treatment, the IRF-8 patterns seen at the promoter level paralleled the IRF-8 patterns observed at the mRNA level. It is interesting to note, however, that since the exogenous promoter fragment did not contain deacetylation sites, these data suggest that TSA could modulate IRF-8 transcription via mechanisms not necessarily related to HDAC inhibition at the promoter level.

We next examined the integrity of events upstream of IRF-8, mainly STAT1 as it is known to be essential for IFN-γ-inducible gene regulation, including IRF-8 [19], [35]. Phosphorylation of STAT1 plays an important role in regulating IFN-γ-mediated gene induction. It has also been reported that HDACi, such as TSA, alters the expression of IFN-γ-inducible genes through acetylation of STAT1 in myeloid cells and tumor cells [42]–[44]. We found that STAT1 silencing in either parental or aggressive CMS4 cells led to a significant reduction in TSA- or IFN-γ-induced IRF-8 promoter activity, the latter of which served as a positive control. These results suggested that TSA-induced IRF-8 promoter activity was STAT1-dependent. However, it is important to emphasize that single agent TSA treatment did not seem to elicit STAT1 phosphorylation, but did promote STAT1 acetylation. Thus, we posit that TSA may impact STAT1 function in an unphosphorylated manner, as previously reported in other systems [36]–[40].

**Figure 6:**
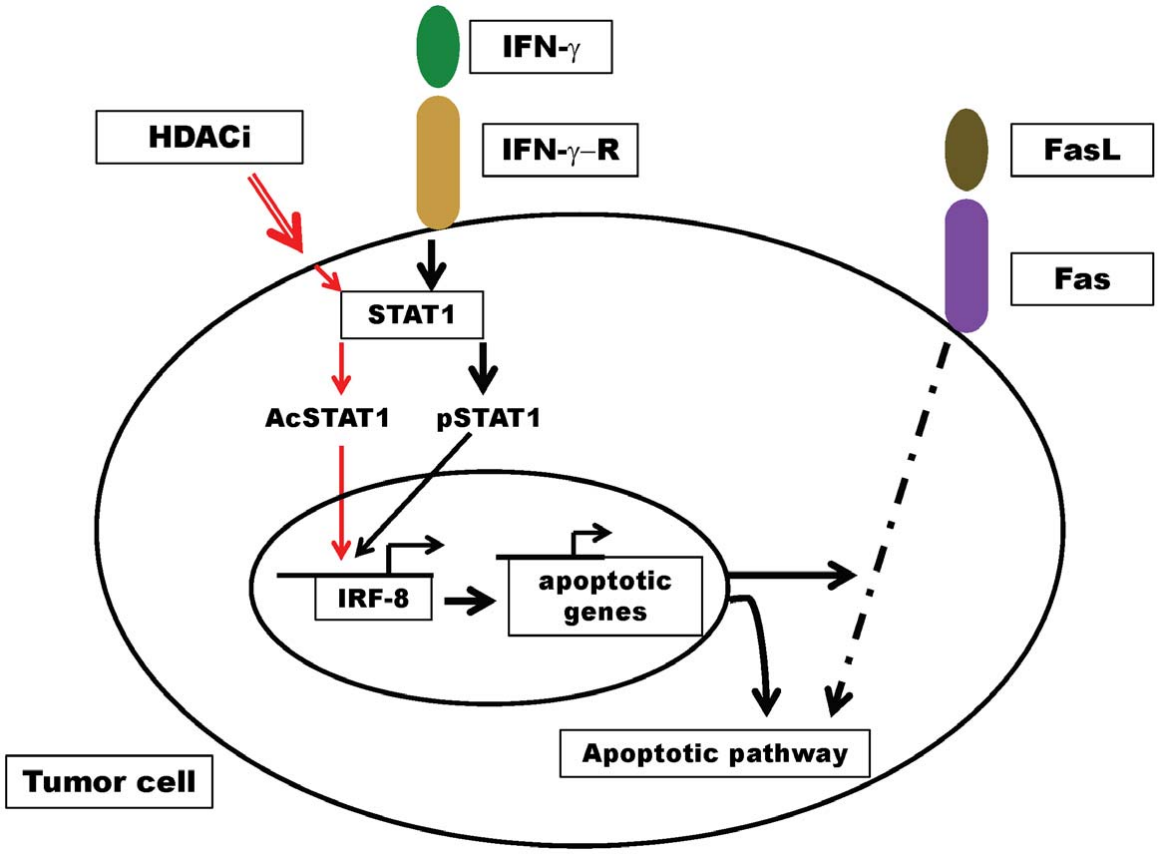
Role for IRF-8 in the HDACi-mediated antitumor response. Engagement of the IFN-γ receptor (IFN-γR) by IFN-γ and/or the uptake of HDACi, such as TSA, induce IRF-8 transcription. While activation of IRF-8 in both cases is STAT1-dependent, the mechanisms by which this is achieved may be distinct and involve phosphorylated and unphosphorylated modifications. In regard to the latter, the precise nature of interactions remains to be fully detailed. IRF-8 expression, in turn, is known to affect apoptosis by regulating genes associated with both extrinsic and intrinsic pathways of cell death (not illustrated).

Overall, our data are consistent with a model that tumor-cell expression of IRF-8 is integral for HDACi-induced antitumor activities (Fig. 6). HDACi exposure may render neoplastic cells more receptive to IRF-8 induction and Fas-mediated death under pro-inflammatory (IFN-γ-dependent) conditions. Therefore, IRF-8 transcription may be influenced in two ways; one by IFN-γ and the other by HDACi (e.g., TSA). In either case, IRF-8 transcription is STAT1-dependent. STAT1 activation, however, may result from both phosphorylation-dependent and –independent (i.e., acetylation) mechanisms, which warrant further study. Moreover, these data do not preclude the possibility that the IRF-8 promoter may be regulated by multiple epigenetic mechanisms, including DNA methylation, and that these mechanisms may impact IRF-8 expression and consequently Fas sensitivity in a direct or indirect manner. Such complex issues, therefore, warrant further study. Nonetheless, the induction of IRF-8, in turn, modulates tumor response to immune attack via Fas-mediated apoptosis. Based on observations in myeloid leukemia, IRF-8 may regulate Fas responsiveness by acting as a transcriptional activator of pro-apoptotic genes, such as caspases, and/or a transcriptional repressor of anti-apoptotic genes, such as PTPN13 (FAP-1) or members of the Bcl-2 family [19], . Altogether, our results point to IRF-8 expression in tumors as being a potential biomarker for efficacy of response to HDACi and a possible molecular target to improve response to therapy.

## Materials and Methods

### Ethics Statement

All experiments were conducted and approved under our Institutional Animal Care and Use Committee at Roswell Park Cancer Institute under protocol ID number 1117M and in accordance with institutional regulations, NIH and Public Health Service policies.

### Cell Lines and Reagents

The mouse sarcoma cell line CMS4 was kindly provided by A. DeLeo (University of Pittsburgh, Pittsburgh, PA) and maintained in culture in RPMI-based culture medium [47]. IRF8-deficient (CMS4-shRNA) or control CMS4 cells were previously [16] generated by transfection with IRF8-specific or scramble shRNA constructs and maintained in culture containing zeocin (2 mg/ml) (Invitrogen, Carlsbad, CA). Control and CMS4 cells expressing a mutant IRF-8 protein (CMS4-K79E) were previously generated [17] by transfection with an empty vector or a dominant-negative mouse IRF-8 construct that harbors a point mutation in its DNA-binding domain (K to E switch at amino acid site 79) [34], respectively. CMS4-K79E or vector control cells were cultured in media containing G418 (0.75 mg/ml) (Invitrogen). We also used a highly aggressive CMS4 subline, termed CMS4.met.sel, which was selected based on resistance to adoptive immunotherapy with tumor-specific CD8^+^ CTL [32]. The human colon carcinoma cell lines SW480 (CCL-228) and SW620 (CCL-227) were obtained from the American Type Culture Collection (Manassas, VA). SW480 and SW620 are two naturally occurring primary and metastatic colon adenocarcinoma cell lines established from the same patient. The SW620 cell line was derived as a lymph node metastasis identified six months later during disease relapse. [33]. Recombinant mouse IFN-γ was obtained from PeproTech (Rocky Hill, NJ). TSA was obtained from Sigma-Aldrich (St. Louis, MO). Depsipeptide (DP) was obtained from the Experimental Therapeutics section of the NCI.

### RT-PCR or Quantitative Real-time PCR Analysis

Tumor cells were seeded in 6-well plates in RPMI-based culture medium. Cells were incubated with either DP (25 ng/ml for 6 hr) or TSA (100 nM –500 nM range for 24 hr) as described [48], [49], followed by addition of IFN-γ (100 U/ml or 200 U/ml where indicated) to specified wells and culture for an additional 24 hr. Total RNA was prepared from treated or untreated tumor cells using a RNeasy mini kit (Qiagen, Valencia, CA) and 1 µg of RNA was used to synthesize cDNA using Superscript II reverse transcriptase (Invitrogen). Amplification of cDNA samples was performed either with Taq DNA Polymerase (Invitrogen) or SYBR Green Master Mix (SA Biosciences, Foster City, CA) according to the manufacturer’s protocol. PCR or qPCR reactions were performed using the following primer sets: mouse IRF-8 (forward 5′-CGTGGAAGACGAGGTTACGCTG-3′ and reverse 5′-GCTGAATGGTGTGTGTCATAGGC-3′), STAT1 (forward 5′-CTTCTTCCTGAACCCCCCG-3′ and reverse 5′-CCCATCATTCCAGAGGCACAG-3′) and β-actin (forward 5′-ATTGTTACCAACTGGGACGACATG-3′ and reverse 5′-CTTCATGAGGTAGTCTGTCAGGTC-3′). Both PCR techniques were carried out as previously described [17]. SYBR green quantification was performed on an ABI7900HT (Applied Biosystems) cycling machine and data analyzed using the ΔΔCT method.

### Cell Death Assay

Cell death was measured by propidium iodide (PI) staining as described [16]. Briefly, tumor cells (2×10^5^ cells/well) were seeded in 6-well culture plates and incubated for 24 hr with TSA (20 nM or 100 nM where indicated), IFN-γ (200 U/ml) or TSA plus IFN-γ (IFN-γ was added 4 hr after TSA treatment). Subsequently, cells were incubated for an additional 24 hr in the absence or presence of recombinant human Fas ligand (FasL; 100 ng/ml; PeproTech). Adherent and suspended cells were collected and treated with PI/RNase solution (Sigma) for 15 min at room temperature and analyzed immediately by flow cytometry. The percentage of cell death was calculated by the formula: percent cell death  =  (percent PI+ cells with FasL) – (percent PI+ cells without FasL). The TSA concentrations (100 nM for CMS4-shRNA cells and 20 nM for K79E cells) chosen for these experiments caused minimal toxicity, as measured by trypan blue exclusion and PI staining.

### Tumor Growth Experiments

BALB/c mice (6–8 weeks of age) were obtained from NCI-Frederick (Frederick, MD) and all animal studies were conducted under IACUC approved protocols. Tumor cells (5×10^5^/mouse suspended in PBS) were injected subcutaneously (SQ) in the ventral trunk of syngeneic mice. Tumor growth was measured twice weekly in two dimensions and tumor volumes were calculated using the formula: volume (mm^3^) = (width^2^ × length)/2. In the TSA treatment experiments, when tumor size reached ∼30 mm^3^, the designated groups of tumor-bearing mice received peritumoral injections of TSA (500 µg/kg body weight) or vehicle (DMSO) in PBS (50 µl) daily for 6 consecutive days, similarly as described [30], [41]. Mice were euthanized when tumor load approached the ethical limit of 2 cm (in either dimension).

### Reporter Assay

The mouse IRF-8 promoter is known to have at least one well-defined palindromic motif (5′-TTCTCGGAA-3′) within positions -175 to -155 for STAT1 binding [50]. An IRF-8 promoter fragment (-257 to -17) that contains this STAT1 binding site was generated by RT-PCR from genomic mouse tail DNA. The following primer sequences were used: full-length forward primer 5′-AGCAGCTAGCGGGTGAGAGTCCTGTAAGC-3′, Core construct 5′-ATCGGCTAGCTCTCCAAACCTGAACGAC-3′ both contains a Nhe1 restriction site. The reverse primer 5′-AGCCTCGAGCGCCTGCTTTTATAGATGG-3′ containing an Xho1 restriction site was for both constructs. The constructs were subcloned into pGL3-basic vector (Promega, Madison, WI). Transfections were performed using Lipofectamine 2000 reagent (Invitrogen) according to the manufacturer’s instructions. CMS4 cells (2×10^5^/well) were transfected with a pGL3 luciferase reporter plasmid lacking or expressing the IRF-8 promoter fragment (1 µg) along with pRL-CMV-renilla (0.1 µg) to normalize for transfection efficiency (Promega, Madison, WI). Where indicated, cells were also co-transfected with mouse STAT1 siRNA or control siRNA (0.6 µg; Santa Cruz, Santa Cruz, CA). At 18 hr post-transfection, cells were treated with TSA (500 nM), IFN-γ (200 U/ml) or both for an additional 6 hr. Subsequently, luciferase activity was measured using the Dual-Luciferase Assay kit (Promega) according to the manufacturer’s protocol. Luminescence was quantified using Monolight 3010 luminometer (BD Biosciences, Franklin Lakes, NJ) and luciferase values were normalized to Renilla using the formula: RLU = [luciferase/renilla].

### Western Blot Analysis

CMS4 and CMS4.met.sel cells were treated with TSA (500 nM) ± IFN-γ (200 U/ml) for 10 to 120 min. Total protein from control and treated cells was extracted with RIPA lysis buffer in presence of protease and phosphatase inhibitors. Protein concentrations were measured using the BCA assay kit (Thermo Scientific, Rockford, IL) and 30 µg of protein/sample was used for gel electrophoresis. The expression of pSTAT1 and total STAT1 was probed using anti-pSTAT1 (1∶800 dilution) or anti-total STAT1 (1∶1,000 dilution) antibody (Cell Signaling, Boston, MA), respectively. Bands were visualized using the Super Signal Western detection kit (Thermo Scientific).

For detection of acetylated STAT1, CMS4.met.sel cells were treated with either vehicle control or TSA (500 nM) for 6 hours. After treatment, cell lysates were recovered by extraction with RIPA buffer (Cell Signaling) containing standard protease and phosphatase inhibitors. Lysates were pre-cleared with rabbit IgG and protein A/G beads (both from Santa Cruz). Protein concentrations were measured by the BCA assay (Thermo Scientific). A total of 800 µg of input protein from each sample was then subjected to immunoprecipitation with rabbit anti-STAT1 antibody (1∶100 dilution; Cell Signaling). The immune complexes were precipitated with the pre-cleared protein A/G beads, boiled and prepared for SDS-PAGE electrophoresis (10% pre-cast gels; Bio-Rad). Acetylation of STAT1 was determined by Western blot using an anti-acetyl-lysine antibody (clone 4G12, 1∶1000 dilution; Upstate/Millipore, Billerica, MA), followed by incubation with HRP-conjugated goat anti-mouse secondary antibody (1∶10,000 dilution; Bio-Rad). Bands were detected using the SuperSignal chemiluminescence detection kit (Thermo Scientific). Band intensities were quantified by densitometry using ImageJ software (NIH), and the data then illustrated as fold-change of TSA-treated vs. untreated samples for acetylated STAT1 or total STAT1 levels.

### Statistical Analysis

For comparisons between control and experimental groups, data were recorded as mean ± SEM of the indicated number of mice or experiments. Statistical analysis was determined using unpaired t-test, two-way paired t-test with Hochberg correction or 2-way ANOVA with Bonferroni post-tests, where appropriate. *P*-values less than 0.05 were considered significant.
